# *Rimbp*, a New Marker for the Nervous System of the Tunicate *Ciona robusta*

**DOI:** 10.3390/genes11091006

**Published:** 2020-08-27

**Authors:** Ugo Coppola, Paola Olivo, Enrico D’Aniello, Christopher J. Johnson, Alberto Stolfi, Filomena Ristoratore

**Affiliations:** 1 Biology and Evolution of Marine Organisms, Stazione Zoologica Anton Dohrn, 80121 Napoli, Italy; Ugo.Coppola@cchmc.org (U.C.); paola.olivo@szn.it (P.O.); enrico.daniello@szn.it (E.D.); 2 School of Biological Sciences, Georgia Institute of Technology, Atlanta, GA 30332, USA; cvjohnson1215@gatech.edu

**Keywords:** bipolar tail neurons, phylogeny, peripheral nervous system, CNEs, enhancers

## Abstract

Establishment of presynaptic mechanisms by proteins that regulate neurotransmitter release in the presynaptic *active zone* is considered a fundamental step in animal evolution. Rab3 interacting molecule-binding proteins (Rimbps) are crucial components of the presynaptic *active zone* and key players in calcium homeostasis. Although *Rimbp* involvement in these dynamics has been described in distantly related models such as fly and human, the role of this family in most invertebrates remains obscure. To fill this gap, we defined the evolutionary history of *Rimbp* family in animals, from sponges to mammals. We report, for the first time, the expression of the two isoforms of the unique *Rimbp* family member in *Ciona robusta* in distinct domains of the larval nervous system. We identify intronic enhancers that are able to drive expression in different nervous system territories partially corresponding to *Rimbp* endogenous expression. The analysis of gene expression patterns and the identification of regulatory elements of *Rimbp* will positively impact our understanding of this family of genes in the context of *Ciona* embryogenesis.

## 1. Introduction

The exocytosis of neurotransmitter-filled synaptic vesicles of nerve and muscle cells is stabilized by various homeostatic signaling systems [1,2,3,4]. Modulation of presynaptic neurotransmitter release relies on an evolutionarily conserved form of homeostatic plasticity in neuromuscular junctions (NMJs), occurring in many distantly-related models ranging from insects to mammals [4,5]. The main mechanisms involved in presynaptic homeostasis are the modulation of Ca^2+^ influx and the regulation of the readily releasable pool of synaptic vesicles. The presynaptic *active zone* contains many conserved proteins such as Rab3-interacting molecules (RIMs), RIM-binding proteins (Rimbps), Munc13, ELKS’s, and α-liprins [6]. Among these, RIMs are likely to be the central organizers that mediate direct or indirect interaction with both the remaining *active zone*-proteins and with those contained in the synaptic vesicles [7,8]. Rimbp proteins interact with Rims1 and Rims2 and are thus important components of the presynaptic *active zone* [9,10] as effectors of the small GTPase Rab3, which is central to regulate the protein composition of the *active zone* [11,12,13]. Furthermore, together with voltage-gated Ca^2+^-channels, Rimbp and RIM proteins are thought to be fundamental in the formation of scaffolds for tethering synaptic vesicles [8]. Rimbps are implicated in the specific control of presynaptic P/Q-type calcium channels through Bassoon [14], confirming that these are crucial players within the presynaptic *active zone* and voltage-gated Ca^2+^ channels. During evolution, Rimbps retained the same domain organization, composed of three SH3- domains and two to three fibronectin III (FN3) repeats [15]. To date, *Rimbp* genes have been studied mainly in vertebrates [9,10,15] with expression in the brain reported both in newborn and adult rats [15]. In mammals, mutations of *RIMBP2* are associated with hearing loss [16] and ones of *RIMBP3* with male infertility [17]. Concerning invertebrates, in the fly *Drosophila melanogaster*, a direct role of Rimbp in modulating the calcium-dependent vesicle release in the active zone [18,19] has also been reported, suggesting a conserved role in synapses.

To shed light on the evolutionary history of these proteins, we performed phylogenetic and synteny analyses of *Rimbp* genes within animals, demonstrating that several duplications have shaped the evolution of this family. To gain insights into *Rimbp* expression in invertebrates, we focused our attention on the sole ortholog of Rimbp proteins present in the tunicate *Ciona robusta*, an invertebrate chordate considered the closest living relative of vertebrates [20]. Whole-mount in situ hybridization (WISH) experiments revealed that during *Ciona* embryogenesis two isoforms of *Rimbp* are differentially expressed in pigment cell precursors (otolith and ocellus) and in cells belonging to the *Ciona* peripheral nervous system (PNS). We showed that *Rimbp* intronic regulatory elements are able to drive expression in papilla neurons, ascending motor ganglion neurons (AMGNs), and bipolar tail neurons (BTNs). Altogether, our results highlight *Rimbp* expression in the nervous system of tunicates such as *Ciona*, paving the way to future studies on the evolution of Rimbp different roles in neurotransmitter release.

## 2. Materials and Methods

### 2.1. Phylogenetic and Synteny Analysis

The amino acid sequences used for the evolutionary survey were retrieved from the NCBI, Ensembl, ANISEED databases, and collected in Table S1, whilst the sequences characterized by a high degree of divergence were listed in Table S2. The Rimbp of tunicate *Ciona robusta* (XP_009858695.2) was the initial query employed for tBlastn [21], and reciprocal Blasts were performed. The obtained protein sequences were aligned using Clustal Omega [22] and the phylogenetic reconstruction was computed with the Maximum Likelihood (ML) estimation using MEGA6 with 1000 replicates and the LG substitution model (γ = 4 and proportion of invariable sites = 0.4) [23], and all the sites were used for the phylogenetic analysis. The graphical representation was carried out with Dendroscope [24]. The syntenic survey of *Rimbp* genes in vertebrates was performed consulting NCBI, Ensembl, and Genomicus databases. The domain architecture of Rimbp proteins in Figure S1 was assessed by using the domain bank of PROSITE database [25].

### 2.2. Animals and Embryo Electroporation

Adults of *Ciona robusta* were gathered from the Gulf of Taranto or from San Diego, CA, USA, (M-REP). Gametes from several animals were taken separately for in vitro cross-fertilization followed by dechorionation and electroporation, as previously described [26,27,28,29]. Embryos were staged according to the developmental timeline established in Hotta et al. 2007 [30]. To visualize GFP, embryos were fixed in MEM-FA (3.7% methanol-free formaldehyde, 0.1 M MOPS pH 7.4, 0.5 M NaCl, 2 mM MgSO_4_, 1 mM EGTA) for 20 min and washed several times in PBS with 0.15% Triton X-100, 0.05% Tween 20. The transgenesis experiments were carried out electroporating 70 micrograms of construct. Each experiment was performed 4 times, and at least 200 embryos were scored.

### 2.3. In Situ Hybridization

Single in situ hybridizations were carried out essentially as illustrated previously [27,28], employing DIG-labeled riboprobes in combination with anti-Dig_AP antibody (Roche, Indianapolis, IN, USA). The antisense riboprobe of the isoform A (800 bp) was PCR-amplified from cDNA of *Ciona* larvae (oligos listed in Table S5) and cloned into P-gem T-Easy vector (Promega, Madison, WI, USA). The antisense RNA probe was transcribed using T7 RNA Polymerase (Roche, Indianapolis, IN, USA) and purified with a Qiagen kit. Instead, the riboprobe of the isoform B was obtained from the Satoh library (plate: R1CiGC06d17), then linearized and transcribed employing T7 RNA Polymerase. The riboprobe of *Rab3Gap1* gene was also obtained from the Satoh library (plate: R1CiGC38o19), next, linearized and transcribed using T7 RNA Polymerase.

### 2.4. Molecular Cloning

The intronic regions of *Rimbp* were selected by using the mVISTA tool of the ANISEED database [31] and PCR-amplified from genomic DNA (sequences are listed in Table S4). The products were cloned into a TOPO-TA vector (Invitrogen, Carlsbad, CA, USA) and then inserted into a vector comprising *eGFP* and human *β-globin* minimal promoter [32]. Oligos used for cloning experiments are listed in Table S5.

## 3. Results

### 3.1. Evolutionary Survey of Rimbp2/3 Genes

**Figure 1:**
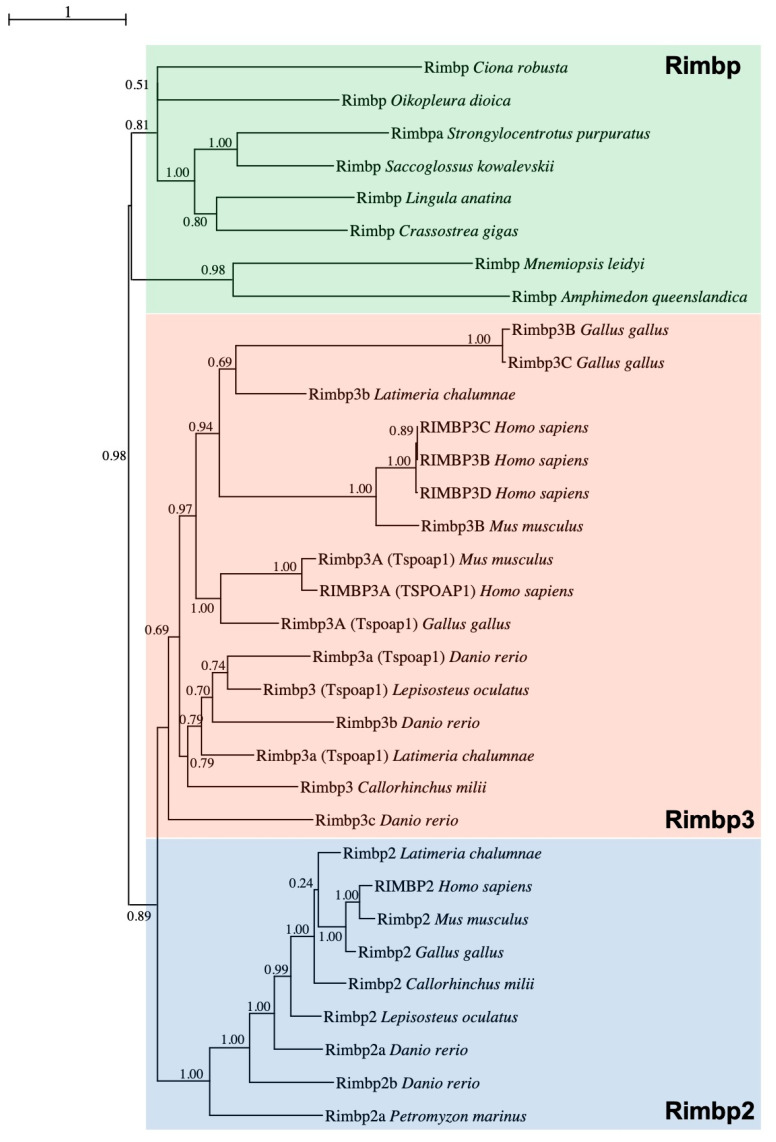
The evolutionary history of Rimbp proteins. Maximum Likelihood (ML) phylogenetic tree supporting the orthology among *Rimbp* of invertebrates (green box) and both *Rimbp2* (blue box) and *Rimbp3* (orange box) of vertebrates, with brackets highlighting the name commonly used for several *Rimbp3*. Values at branches represent replicates obtained using the ML estimation method; the whole protein sequence has been used for tree inference.

In order to clarify the orthology of a gene previously named *Bzrap* (benzodiazepine receptor-associated protein) [28], found to be FGF-regulated in *Ciona* pigmented cells, corresponding to the KyotoHoya gene model KH.C5.558 [33], we performed a Maximum Likelihood (ML) phylogenetic reconstruction (Figure 1) using a manually curated database (Table S1).

In vertebrates, TSPO Associated Protein 1 or Tspoap1, also known as Rimbp1, has previously been referred to as Bzrap due to its ability to interact with the peripheral benzodiazepine receptor of mitochondria [34]. Within the tunicate-specific database ANISEED [31], reciprocal BLASTs revealed that the previously named *Bzrap* gene of *C. robusta* was closely related to various *Rimbp2/3* family genes, for this reason, it has been called *Rimbp* Domain analysis confirmed that *Ciona* Rimbp protein shared the domain organization based on FN3 repeats and Src homology 3 (SH3) (Figure S1). To decipher *Rimbp* evolutionary history, we improved a previous analysis of *Rimbp* genes [15] by including in our survey proteins from Porifera up to Chordates. More specifically, we analyzed sequences belonging to the following species: the sponge *Amphimedon queenslandica*, the ctenophore *Mnemiopsis leidyi*, the cnidarian *Nematostella vectensis*, the brachiopod *Lingula anatina*, the arthropods *Drosophila melanogaster* (insect), *Anopheles gambiae* (insect) and *Daphnia magna* (crustacean), the nematode *Caenorhabditis elegans*, the mollusks *Crassostrea gigas* (bivalve) and *Octopus bimaculoides* (cephalopod), the ambulacrarians *Saccoglossus kowalevskii* (hemichordate) and *Strongylocentrotus purpuratus* (echinoderm), the tunicates *Oikopleura dioica* (larvacean) and *Ciona robusta*, *Phallusia mammillata*, *Halocynthia aurantium* (ascidians), the vertebrates *Petromyzon marinus* (lamprey), *Callorhinchus milii* (cartilaginous fish), *Latimeria chalumnae* (coelacanth), *Lepisosteus oculatus* (non-teleost actinopterygian), *Danio rerio* (teleost), *Xenopus tropicalis* (amphibian), *Podarcis muralis* (reptile), *Gallus gallus* (avian), *Mus musculus* and *Homo sapiens* (mammals) (Table S1). Sequences characterized by a high degree of evolutionary divergence have been excluded from the phylogenetic study (Table S2). We did not find any *Rimbp* ortholog in unicellular eukaryotes and plants. This evolutionary survey based on 48 Rimbp proteins strongly supports the existence of a single *Rimbp* in tunicates and other invertebrates (green box) that resulted in being orthologous to both vertebrate *Rimbp2* (blue box) and *Rimbp3* (orange box) (Figure 1). Among the surveyed invertebrate species, sea urchin *S. purpuratus* was the only one showing duplication (here, referred to as *Rimbpa* and *Rimbpb*), with Rimbpb excluded from the tree given its evolutionary divergence (Table S2). The vertebrate protein classes named Rimbp2 and Rimbp3 (Figure 1 and Table S2) encompass various proteins that in genome databases (Ensembl, NCBI) are named in a different manner (see Table S3 for old and new names). Our phylogenetic reconstruction clearly demonstrated that the human protein known as Tspoap1 belongs to Rimbp3 cluster (Figure 1), thus we renamed it as *RIMBP3A*. Moreover, the human gene known as RIMBP3 has been renamed *RIMBP3D* (Figure 1 and Table S3). This analysis allowed the identification of several *Rimbp2* and *Rimbp3* duplicates in distantly related vertebrates such as zebrafish, coelacanth, and human (Figure 1 and Table S2). The existence of *Rimbp* duplicates could have resulted from isolated cases of gene duplication or from one of the two whole-genome duplications that occurred at the stem of vertebrates [35,36]. To better understand *Rimbp* evolution in chordates, we surveyed the conservation of the *Rimbp* genome environment (Figure S2), allowing us also to define the orthology of vertebrate *Rimbps* better. In vertebrates, *Rimbp2* genes belong to a region comprising a conserved linkage formed by *Ran* and *Stx* genes [37], which is not syntenic with *Rimbp3* genomic locus (Figure S2). These findings suggest a local duplication at the root of vertebrates involving the ancestor of these two genes. Interestingly, ascidian *Rimbp* (*C. robusta*, *Halocynthia aurantium*) and vertebrate *Rimbp3* genes preserved chromosomal vicinity with the mitochondrial E3 ligase *Mul1* [38]. Furthermore, in the tunicates *Ciona robusta* and *Phallusia mammillata, Rimbp* forms a triplet (Figure S2) with *Rab3Gap1*, which in vertebrates is implicated in Warburg and Martsolf syndromes and, like Rimbp proteins, is an effector of Rab3 proteins [39,40,41], and *Chmp2* [42]. In synthesis, our evolutionary data strongly support an orthology of ascidian *Rimbp* with the invertebrate *Rimbp* and with vertebrate *Rimbp2* and *Rimbp3* genes, which are affected by diverse duplication events.

### 3.2. Dynamic Expression Pattern of Rimbp in the Ciona robusta Nervous System

**Figure 2:**
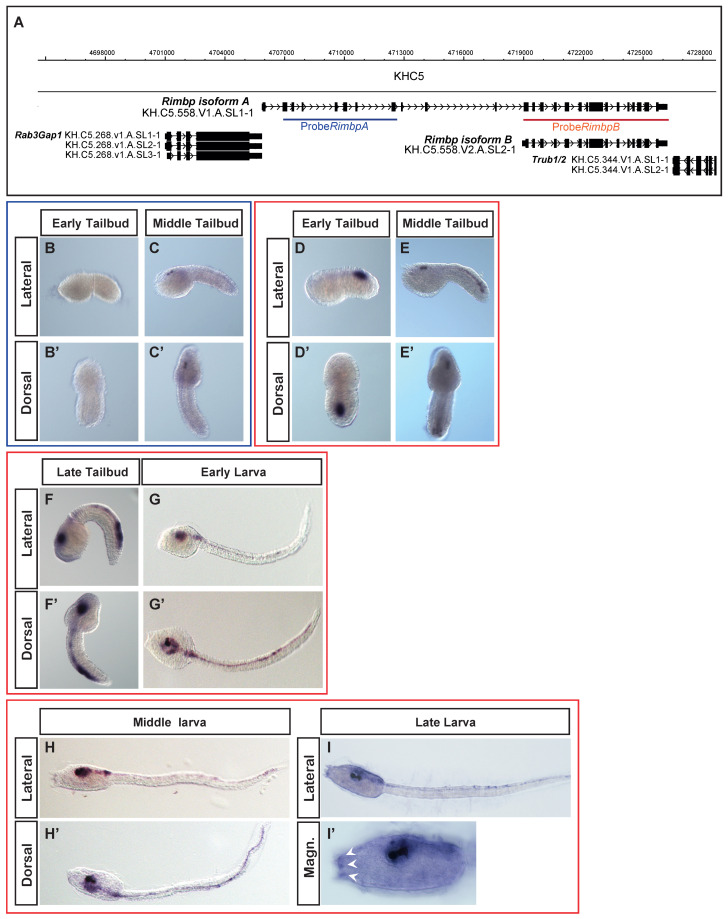
*Ciona robusta* Whole-mount in situ hybridization (WISH) of *Rimbp*. (**A**) Genomic organization of isoform A (blue) and isoform B (red) of *Rimbp*. (**B**–**C′**): *isoform A* is expressed only in the sensory vesicle at the middle tailbud stage (**C**,**C′**). (**D**–**I′**) *isoform B* expression. At the early tailbud stage, this isoform is expressed in the posterior part of the developing nervous system (**D**,**D′**) while from the middle to late tailbud, expands its expression in the sensory vesicle (**E**–**F′**). At early and middle larva stages, *isoform B* is also expressed in the sensory vesicle and motor ganglion (**G**–**H′**). At the middle and late larva, the *isoform B* starts to be expressed in the adhesive papillae organs (white arrowhead **H**–**I′**).

To garner an understanding of *Rimbp* expression in invertebrate chordates, we investigated its expression pattern during *Ciona robusta* embryogenesis by whole-mount in situ hybridization (Figure 2).

Our previous data showed that this gene was expressed in pigment cell precursors at the middle tailbud stage [28]. A more detailed analysis of the genomic locus using the ANISEED database showed that *Ciona Rimbp* exhibits two different transcript isoforms, the longer one measuring 4.7 kb (isoform A) and the shorter that measures 3.3 kb (isoform B). More in details, we found that the isoform B does not include the first 11 exons of the isoform A (Figure 2A), although both isoforms encompass the SH3 and FN3 domains. Moreover, the isoform A-unique exons encode an N-terminal protein sequence that is present in human RIMBP3 proteins, but not in RIMBP2 (Figure S1).

To distinguish between the expression of two isoforms, we synthesized two probes: one encompassing only the first region of the isoform A (blue) and specific for this isoform, the other comprising all of the isoform B (red), thus potentially able to recognize both the isoforms (Figure 2A). Isoform A was expressed exclusively at the middle tailbud stage in the sensory vesicle with specific expression in two cells corresponding to ocellus and otolith pigment cell precursors (Figure 2B–C′), as shown in our previous survey [28]. Regarding the isoform B, we found that its expression starts during the early tailbud stage in the posterior part of the developing nervous system, in what appears to be A8.16-derived ependymal cells [43] (in the lateral rows of the neural tube (Figure 2D,D′). As development proceeds, from the middle to late tailbud, we found expanded *Rimbp* isoform B expression in the sensory vesicle, indicating expression in larval brain neurons (Figure 2E–F′). In the posterior region, the expression also expanded to include the bipolar tail neurons (BTNs) [44], which have been compared to dorsal root ganglia neurons (DRGNs) of vertebrates [45]. Between the late tailbud and early larva, we also detected expression in the motor ganglion. At the early larva stage, the isoform B continued to be expressed in neurons of the brain, motor ganglion, and tail (Figure 2G,G′). Interestingly, at the middle and late larva stages, we detected expression also in the adhesive papillae organs (Figure 2H–I′). Due to overlap of probe B with both isoforms, we cannot say if the detected expression in the pigmented cell precursors (Figure 2E,E′) is specific of the isoform B or results from the isoform A expression.

Our characterization of *Rimbp* expression pattern in tunicate *Ciona robusta* demonstrated, for the first time, that *Rimbp* isoforms have differential expression patterns and activation time. Indeed, isoform B shows a strong expression in different areas of the nervous system starting from the early tailbud up to the larva stage, while isoform A is transiently expressed only in the pigment cells at the middle tailbud.

### 3.3. Intronic Cis-Regulatory Elements for Rimbp Expression

**Figure 3:**
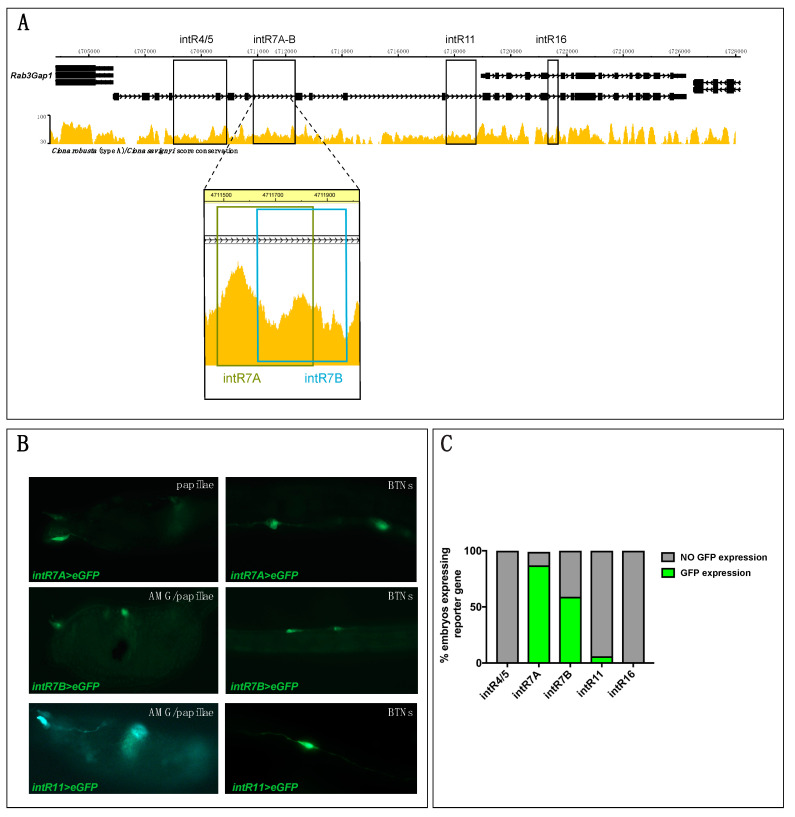
Intronic *Cis*-regulatory elements of *Rimbp* of *Ciona robusta*. (**A**) The genomic region comprising *Rimbp* and *Rab3Gap1* with an automatic mVISTA plot between *C. robusta* and C. *savignyi* (ANISEED); rectangles indicate the intronic regions selected for cloning in *eGFP* vector (*intR4/5*, *intR7*, *intR11, intR16*). Dashed lines show a higher magnification of the two distinct regions selected for the *intR7* (*intR7A* and *intR7B*). (**B**) Expression of larvae electroporated with 70 micrograms of *intR7A*>eGFP, *intR7B*>eGFP, and *intR11*>eGFP in different PNS territories of larva (st. 26) possibly including BTNs, papillae neurons, and AMGNs. (**C**) Percentages of larvae electroporated along with *intR4/5*, *intR7*, and *intR11*, *intR16* expressing with GFP in at least one type of cells of the nervous system. Each experiment was performed four times and at least 200 embryos were scored per each experiment.

In light of *Rimbp*’s dynamic expression in the nervous system, we focused on its regulatory logics during development (Figure 3). However, the identification of a “canonical” promoter or upstream regulatory region(s) responsible for *Rimbp* expression was not possible because this gene is located in close proximity to the *Rab3Gap1* gene (Figure 3A) in an arrangement that suggests the formation of a putative “two-gene” operon [46].

**Figure 4:**
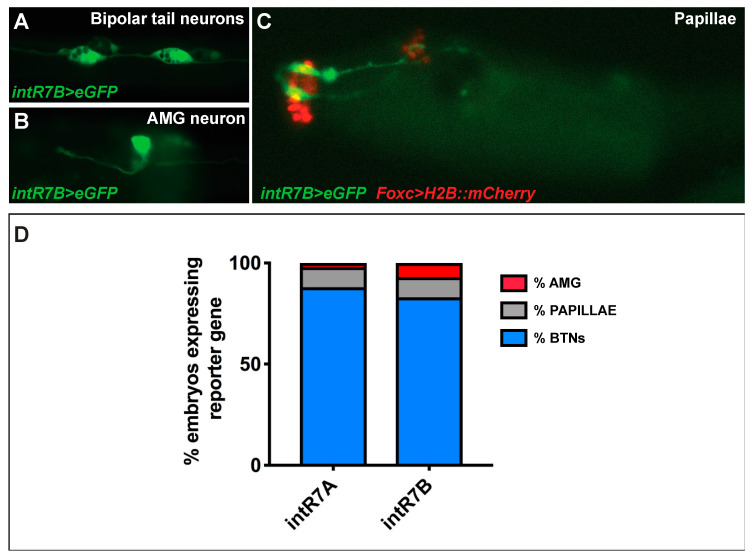
*intR7>eGFP* in PNS of *Ciona robusta*. (**A**) GFP expression in bipolar tail neurons (BTNs). (**B**) Expression in the neurons of ascending motor ganglion (AMG). (**C**) Co-expression of intR7B>GFP and FoxC>H2BmCherry (for both we electroporated 70 micrograms) in glutamatergic neurons of palps. (**D**) Percentages of larvae expressing *intR7A* and *intR7B,* highlighting the relative expression in distinct nervous system territories. Each experiment was performed four times, and at least 200 embryos were scored.

We tried to isolate the regulatory region upstream of *Rab3Gap1*, but despite several attempts, we did not succeed, possibly due to problems in this genomic region. Therefore, to find regulatory regions underlying *Rimbp* expression, we took advantage of the mVISTA tool available on ANISEED to identify highly conserved non-coding elements (CNEs) with the sibling species *Ciona savignyi* within *Rimbp* introns (Figure 3A and Table S4). We cloned four conserved CNEs upstream of a GFP reporter gene [32], respectively, named *intR4/5* (1.8 Kb), *intR7* (0.3 Kb), *intR11* (0.7 Kb), *intR16* (0.3 Kb) (Figure 3A). Specifically, for *intR7* region, we isolated two partially overlapping fragments named *intR7A* and *intR7B* (Table S4 and Figure 3A). The selected fragments were electroporated in *Ciona* eggs to investigate their capability to drive expression of the reporter gene in the same territory of the endogenous transcript. Results at the larval stage showed that the *intR7A>eGFP, intR7B>eGFP,* and *intR11>eGFP* were able to drive expression in some cells of the peripheral nervous system (PNS) (Figure 3B,C), possibly including BTNs, papillae neurons, and AMGs. In contrast, *intR4/5>eGFP* and *intR16>eGFP* did not show any GFP signal (Figure 3C). Both *intR7A* and *intR7B* constructs drove a strong expression in the PNS of a majority of electroporated larvae, whilst *intR11>eGFP* expression signal was reported in the PNS of only a few embryos (less than 10%) (Figure 3C). Even though the *intR7* constructs encompass overlapping regions, the *intR7A>eGFP* expression showed a significantly higher rate in terms of larvae expressing GFP (Figure 3C). Our analyses, however, did not reveal a strong positive signal of the reporter gene in the pigmented cell precursors, brain neurons, or ependymal cells of the tail, as shown for the endogenous transcript (Figure 2) suggesting that active motifs for this territory are not all present in the isolated regulatory elements. To better understand the *intR7*>*eGFP* expression in various neurons of the *Ciona* PNS at the larva stage, we analyzed, in better detail, the larvae electroporated with *intR7B>eGFP* (Figure 4).

As for the endogenous signal, we observed a strong expression in BTNs, [44,47] (Figure 4A,D). These neurons are situated on either side of the neural tube and extend their axons along the entire length of the tail. Electroporated larvae also showed strong expression in ascending motor ganglion or AMG neurons (AMGNs), which are peripheral interneurons located dorsally to the core motor ganglion [47] (Figure 4B). The *intR7* guided expression, also in neurons of the papillae, which are glutamatergic neurons that project their axons along the rostral PNS and into the sensory vesicle [48]. Papilla neuron expression of intR7 was confirmed by double electroporation along with *FoxC>H2BmCherry,* which marks the papilla territory [48] (Figure 4C). Hence, we found that both *intR7A* and *intR7B* drive expression in the same nervous territories with the signal in BTN cells represented the vast majority, with 80% of positive larvae (Figure 4D).

In summary, although we were not able to identify the regulatory region(s) responsible for the whole endogenous signal, we discovered three *Rimbp* intronic enhancers active in the PNS of *Ciona robusta* (*intR7A, intR7B, intR11*).

## 4. Discussion

Since the evolution of *Rimbp* genes is not well-known, we reconstructed the history of this family in animals. Rimbp proteins are considered to represent a fundamental tool for the establishment of presynaptic machinery in metazoans [49,50]: This speculation is in agreement with *Rimbp* absence in plants and the majority of unicellular eukaryotes but the presence in choanoflagellates (*Monosiga brevicollis*) and in non-bilaterian animals (sponges, ctenophores). The high degree of conservation of *Rimbp* genes in all the animals is coherent with their conserved role in presynaptic protein dynamics. Our genomic survey showed that invertebrates possess a single *Rimbp* gene, with the only the exception of the sea urchin exhibiting an independent duplication, whilst gnathostomes have *Rimbp2* and *Rimbp3* duplicates (Figure 1, Table S2 and Figure S2). The concept of orthology among invertebrate *Rimbp*, *Rimbp2,* and *Rimbp3* genes is enforced by the synteny we found between human *RIMBP3A* and *Rimbp* of ascidians (Figure S2). Moreover, a strong phylogenetic signal proved that *Rimbp* genes are preserved in all the surveyed tunicates (Figure 1 and Figure S2), despite these animals had undergone massive gene losses [51,52], suggesting an essential role for *Rimbp*.

Although the *Rimbp2* and *Rimbp3* loci are conserved among gnathostomes ([37]; Figure S2), there is lack of synteny between them and in the lamprey (considered an ancestor of gnathostomes) we found only two *Rimbp2* genes (Figure 1 and Table S2). Therefore, we suggest that *Rimbp2* and *Rimbp3* orthologs derived from a gene duplication event occurred at gnathostome evolutionary radiation, as strongly suggested by the presence of a single copy of both *Rimbp2* and *Rimbp3* in cartilaginous fish *C. milii* and in non-teleost fish *L. oculatus* (Figure 1). The topology of the tree and the syntenic analysis evidenced a stable evolutionary history for the *Rimbp2* lineage and major diversification for *Rimbp3*, with distinct origins for several vertebrate paralogs. Because the relationship existing among the various *Rimbp3* duplicates was not clarified using BLAST and phylogeny, synteny analysis permitted us to define the orthology of *Rimbp3* genes (Figure S2). In fact, *Rimbp3a* and *Rimbp3b* conserved in coelacanth and zebrafish do not correspond to human *RIMBP3A* and *RIMBP3B* (Figure 1 and Figure S2): The former derives from a tandem duplication, the latter from an independent gene duplication event. Importantly, the presence of one *Rimbp3* gene in amphibians, two *Rimbp3* in reptiles, and three *Rimbp3* genes in chicken (Figure 1 and Table S2) lead to hypothesize the existence of three duplicates in the ancestor of tetrapods (with the fourth possibly emerged in primates). Another explanation is that *Rimbp3* duplication has occurred during amniote evolution. Phylogeny and synteny data speak in favor of a specific duplication event followed by a double tandem duplication with the successive loss of the fourth member. Otherwise, the presence in mouse and other mammals (dog, pig) of only two *Rimbp3* genes suggests further lineage-specific losses. The additional member of zebrafish (*rimbp3a2*) possibly arose from teleost specific whole-genome duplication (TSGD) or 3R [53,54], as indicated by the retained synteny on chromosomes 5 and 15 (Figure S2) conserved in other teleost genomes as golden-line barbel, goldfish (NCBI, Ensembl). Moreover, the presence of *Syntaxin2* (*stx2*) orthologs close to both zebrafish *Rimbp2* gene *loci* (Figure S2), lead us to hypothesize the same origin for them. Among tetrapod *Rimbp3* genes, the most ancient member is *Rimbp3A*, as testified by its conserved synteny and the partial preservation with the *Rimbp* genome environment of ascidians (Figure S2). Moreover, if we consider the whole-genome duplications involving vertebrates, the current number of *Rimbp* repertoire in vertebrate models clarifies that many *Rimbp* orthologs have been lost. Thus, *Rimbp* evolution was shaped mainly by gene duplications and massive losses (in particular *Rimbp3*). Discovering new insights about *Rimbp* expression and functions in other vertebrate model systems will be important in understanding the impact of genomic rearrangements on the evolution of presynaptic functions.

In mammals, while RIMBP2 is associated with presynaptic functions [14,55,56], RIMBP3 proteins seem to have a role in microtubule organization, especially in spermatozoa [17,57]. Possibly, these different biological roles can be associated with their divergent domain architecture (Figure S1). In *Ciona*, two different isoforms of Rimbp have been found: Both share the same domain structure (Figure S1) and the genome environment (Figure S2) with human Rimbps3 but the shorter *Ciona* isoform lacks the N-terminus region specific of RIMBP3 proteins. It is tempting to speculate that in invertebrate chordates *Rimbp* played multiple functions through alternative transcripts. Then, some ancient sub-functionalization could have been cemented genetically through the various duplications occurring in vertebrates. In light of domain organization of *N. vectensis*, the most parsimonious explanation is that the Rimbp ancestor possessed three FN3 motifs, with high degree of variability among metazoan Rimbps. In particular, we registered distinct FN3 losses in various species, with the dramatic case of total absence in *C. milii* Rimbp3.

To gain information on this family in invertebrates, we analyzed, for the first time, the expression and the regulation of the sole *Rimbp* in the tunicate *Ciona robusta* (Figure 2 and Figure 3). In situ hybridization of *Rimbp* during *Ciona* embryogenesis revealed a substantial difference in the expression pattern between isoform A and B. The first is active only in pigment cell precursors during the early and middle tailbud stage, while it does not show any expression at the larva stages, suggesting a transient expression. The isoform B instead is expressed in cells of the central and peripheral nervous systems starting from the middle tailbud stage until the larva stages (Figure 2). Due to the fact that probe B is not able to distinguish among the two isoforms, we cannot say if the isoform B is also expressed in pigment cell precursors or not.

Regarding the regulatory mechanisms underlying *Rimbp* dynamic expression, we found three intronic regions (*intR7A*, *intR7B, intR11*) driving expression in neurons of the peripheral nervous system, even if with different efficiencies (Figure 3). These regions are conserved between *Ciona* sibling species, but no preservation with other tunicates and/or vertebrates has been observed. We did not find any regulatory element able to drive expression in the central nervous system (brain and ependymal cells of the tail) nor in the pigment cell precursors, suggesting that additional regulatory regions remain unidentified. Where the regulatory region specific for the isoform A is located remains not clear due to close proximity of another gene, *Rab3Gap1*, which expression is not detectable employing WISH and forming a putative operon together with *Rimbp*. Traditionally considered as a prokaryotic characteristic, operons have been proposed to be a specialized feature of the unusually compact *Ciona* genome [58]. Intriguingly, like Rimbp proteins, Rab3Gap1 is implicated in the regulation of Rab3 in the context of presynaptic dynamics [59]. Although operons in *Ciona* do not necessarily encompass genes with similar functions [58], the fact that both Rab3Gap1 and Rimbp proteins are functionally related in the cell, together with their chromosomal vicinity, could suggest common gene regulation modality [60,61]. Moreover, the preservation of *Rab3Gap1-Rimbp* duplet (Figure S2) within *P. mammillata* (Figure S2) and the genome conservation between *C. robusta* and *P. mammillata* (ANISEED browser) in this region, which evokes similar genomic organization in both the species.

Interestingly, the specific expression of isoform A in the cells of pigment cell lineage and its similarities with *RIMBP3* organization evokes the possibility that *Rimbp* of *Ciona* has similar cellular functions of mammalian Rimbp3. It is alluring to speculate that they have a common role in the stabilization of microtubules, which could have a role in the formation of *Ciona* pigment cell structure. Consequently, it would be significant to gain insights on the *Rimbp* functions in *Ciona* and on the role in embryogenesis of *Rimbp* orthologs in other invertebrates. The expression of *Rimbp* in most of the *Ciona* larval nervous system suggests this gene encodes an important effector of neuronal function in this invertebrate chordate.

In sum, our results confirm the putatively conserved role for Rimbp proteins in neuronal presynaptic function and provide insights into the potential role of *Rimbp* gene duplications and subfunctionalization in the evolution of the vertebrate nervous system.

## Supplementary Materials

The following are available online at https://www.mdpi.com/2073-4425/11/9/1006/s1. Figure S1: Scheme of domain organization in metazoan Rimbp proteins; Figure S2: Comparison of genomic loci of three ascidians; Table S1: FASTA encompassing the Rimbp protein sequences used for the phylogenetic tree of Figure 1; Table S2: FASTA with the Rimbp protein sequences found using Blast and excluded from phylogeny of Figure 1 for their divergence; Table S3: List of employed Rimbp sequences, with old and new names, according to phylogenetic and syntenic data; Table S4: Intronic sequences with relative chromosomal positions that have been cloned in *eGFP* vector and tested via electroporation; Table S5. List of oligos used for cloning experiments.
