# Supplementary material for: Rimbp, a New Marker for the Nervous System of the Tunicate Ciona robusta
Source: Genes (Basel). 2020 Aug 27;11(9):1006. doi: 10.3390/genes11091006 (PMC7565545; doi:10.3390/genes11091006)
Supplement: Supplementary file 1 [file genes-11-01006-s001.zip › coppola et al. Table S4.docx]

**Table S4.** Intronic sequences, with relative chromosomal positions, that have been cloned in *eGFP* vector and tested *via* electroporation.

**>intR4/5_KhC5:4708078-4709888**

GCTCCTATGTTTTGCAGGCATTTTCACTATATGTATATTATGTTAAACTTTTGGCAATTTTGTAACTGGGTTCTTAAAGGACCGACCTTTAGACGTTCTATTCTGGCCCCCTTTATCGGGGTATGTCATTGAGTGTCATGTCCAAGTACCGATAAAGATGATCGGAAAAGGCGTCTAAATGTCAATCCTTTCAGATCAGTGTTCCAACTGCCCGAAGATCTCCTTCAGAATTTATTAACATAGTAAAAAAAAGTTCTTTAAAATAACTTTGTATATATTTCCTTTTAATTTATCTTTTATATTTCACAATTCGTATACGTTGGTCAAAAAATGTTCTATTTTCCTGCCCTACCATGTTCTAAACTAGGGCTTTCCAAACTAGGGGGTAAAGAGACAAAATACTACCTACAATATACTATTAAGGGCCTTGGAAAATTTTAGACGATTAAAAGAGGCCGCATGCAAAAGTTTGGGAAGCCCTGTTTTTTGTTCTACCTGTTTAGTAATTTAAAACACTCACAATTTTATTTCTTCCCTTCTGCCCTCTTTGATTAGTCATTTGGTAAGTCTTGTTTTAATGCTTGGTAATCACTTGCATATGAAATTATATGCATGGTGTTATGAACTTATGATGCAACTTTGAAACTTCTATTTAATTCTGCATGTATGATGGGTCAACAGGAATGAGCCAGTACAGTGATTTTCAACCAGTGTGTATACAGTATAAAGTATAACTATGTATAAGTAAATGCTATAAAATAGAAACATGATACTAATAGTTAGTATGTGTATAAATGGCTTATTATTTTACCACATAATTTATTTAAACGAGTTTTTGGCGTAAACTTTCCTTTGTCATTTTTGTACCACATACTTTTTAAGGTAAGAAACTCCTATAAAACATAGTAAACACGGAACGGTTATTATGTTTAATAACTCCTATGTAAACTTTTTTTTATAGTTACATATGCCTTTCTACATATCAGGTAGAAAATCACTGTTCTGTTATATTATATAATTATGCATGAAAATAATTACTATTCACAAAACTTAATGCATTGCTGTGTTACTTGCTTTGGGATTTTCAATACTAATTCAACCTATTTTGGGTTATTTCTTGTAATTAATCATTTTATGTTGTTAAAGTTATGTGCTGTGTGCAACATTGCTACAATCCCTGCCAGTTAAACAGTGGCACTTTTTTTGTCTCTTTGCCCAATTAATTAAGTGTCAGTGATGTAATTAAGTGTATTGCAGTAATAGCTAAAATTCTCCTAATTTTCATTAATAATCTTTTACACTGTATATATATTAAAATACAGCAGAAAATTAAACAAATCCAGTTTAAAATTGTATGCGCCCACCCCCAAAAAAAACCTTAACCATAAGTTGTACACAGTAAATATATATTTCCTAGACTAAGACATTTTCCTTTATAACTAACCCACCTAACCCAAATCTTCCAGCCACGTACGGATCGTCAGTTAAAATACGAGGAGAGGATAATCGATCTGAAGAAGAAGAACACGGAACTTGTAGCGATTGCGAGAAGATTGGAGGAAAAAGCAAAATCCCTGCAAGAGCAAAATGTTAAGGTAGTTCTATTTGTTTTTAGTGTTTCACACATATATATATACAATTTTTCATGTGTTTATACGTTTTTACACTTTTTAAATAGTAGGTTTTTACAAATATATATATATATCAGTTTCACATATACTACCCATTTAAAATTGACATGTAGAACAGCTAAATTGTCAGCCATACATTAAAAAAGAGAGTTAAATATAAGGTAACTTGTAAGCGGGCAC

**>intR7A_KhC5:4711478-4711847**

AAGATTTCAACCATTGATAAGATTTTGTTAAAAATCAATATTCATTATTTTTTTCTCAGCTTTATTTATCCATCAGTCTTCCATTAACCAGATGCCTGTCATTAATACGGGTGAAAAAGGGGAAGCAGCGTTAATAAATATTTTATCAGTGTGCTCGTGCCAGTCTGGTCCAGCATAGTTTTCAGCACTTCAATTTGAATGTGAATGTTTGAAAATTTTGTGGTAGTAGTTTAGATGCAAATTTTGTAGTTATGTGTTAAATTTTAGATTTTTTGTAACTTTTAGTTTAAAAATTAAAACGAATTAATAAGGTTTTAAAATATTTTTTTTATTATTTAATTTTTCGTAATTTTCAGTTGCAAAATTAGGA

**>intR7B** _**KhC5:4711665-4711999**

CTTCAATTTGAATGTGAATGTTTGAAAATTTTGTGGTAGTAGTTTAGATGCAAATTTTGTAGTTATGTGTTAAATTTTAGATTTTTTGTAACTTTTAGTTTAAAAATTAAAACGAATTAATAAGGTTTTAAAATATTTTTTTTATTATTTAATTTTTCGTAATTTTCAGTTGCAAAATTAGGATTTTTTTTATAAATTGGTTTTAGTTAAAAAATTTAGGCTAAAATGGTTAAGCGATTCAACTTTTGGTGATTATTGTAGTGCCTCAAAATAATAGGCAAGATTAATTTACATGTTATGTTTGTGTTTCAATACACTAAATATCTTTGCAATAA

**>intR11_KhC5:4718083-4718844**

GAATTGGAAACGACGACGTTTTTTATGTTTAATTATTCATTAGTATAAATATTAATGCAAAAAAAAGCTGCTTGGTTTTACCACGATGCAATTTTAGACCTTGGTGTCTCATTTATTTTGAACCACGTGCATTACCCACAAGAGTCAATTACAAATCCTGGCGGCATTTATTGATTTTTCCGTGACTGTCAAGCCGTTGCCTATTCCTGCCTGTGCAGAACGCTATTGCCTAATCGGGTGTAAGGATGCCAGGAGATGATTAAGTTTTAGGGAACTTGTTGTGGTTGTGTAATTATGTTACACCATTAGCTTAGAAGGTCGAGGCTTAGTGTAGGGTGGCAGCTAATATAGACATGCATAGTTGGAGTTATAGGAAAATTTAGACATACAATATAGACATACATAGGTACATTACTCGCTGTGAACATTGTGTAACATTTGCAGAAGAAGTTTATAGATTAAGGATTAAATGTCAGACTATATAAGGTGTATGAAATATTGTTTTTACCTTGTCCTATAATATGTCCTATCAATATGGTTTTAGTATTGGAAATGTGTCCATTATGAAATGTAGTATAAGTTATAGATTTTACAAAGATTGGAATACAAAAAGGTGTTCAGTAGTAATACATGTATAAAATACTGTGTTTCGTAAAACATCAAATCAGTGCAGTAATGATTGAGTTTTAAAGGGAGACTATTCCTATTAAAAGATTGTAGCTATTTTCGTCTAATATTCTTGTTGCTAAATAGATTCTAATG

**>intR16_KhC5:4721360-4721676**

TGTTTTTTGGTAAAGCGCATGTTTGGGTTGTATTTAAAGTGTTTATTTATATTTAAAAACCATTTAAAACAGTGGTCGAAAGGCTTTACACAGTCCTATTGTTCTAAATCATGGTTTTAAAGTAAAACAATAGGGGACAAAGTTTAGGCAAGTACAGTTAGCCTAAAAAGTTTTACTTTTCTAAATATCAATTATAAAGACTAATACTTTCCAATCAATAATGTTTTTCTCTTTATATAAAGTACATAGGTAATAGCTTAGTGTATACTGACATAATGCTAAATTAATACTAAGTTTAAACTAGTATTGCAGCCTTC
