# Supplementary figures and images for: Rimbp, a New Marker for the Nervous System of the Tunicate Ciona robusta
Source: Genes (Basel). 2020 Aug 27;11(9):1006. doi: 10.3390/genes11091006 (PMC7565545; doi:10.3390/genes11091006)

**Figure S1.** Scheme of domain organization in metazoan Rimbp proteins.

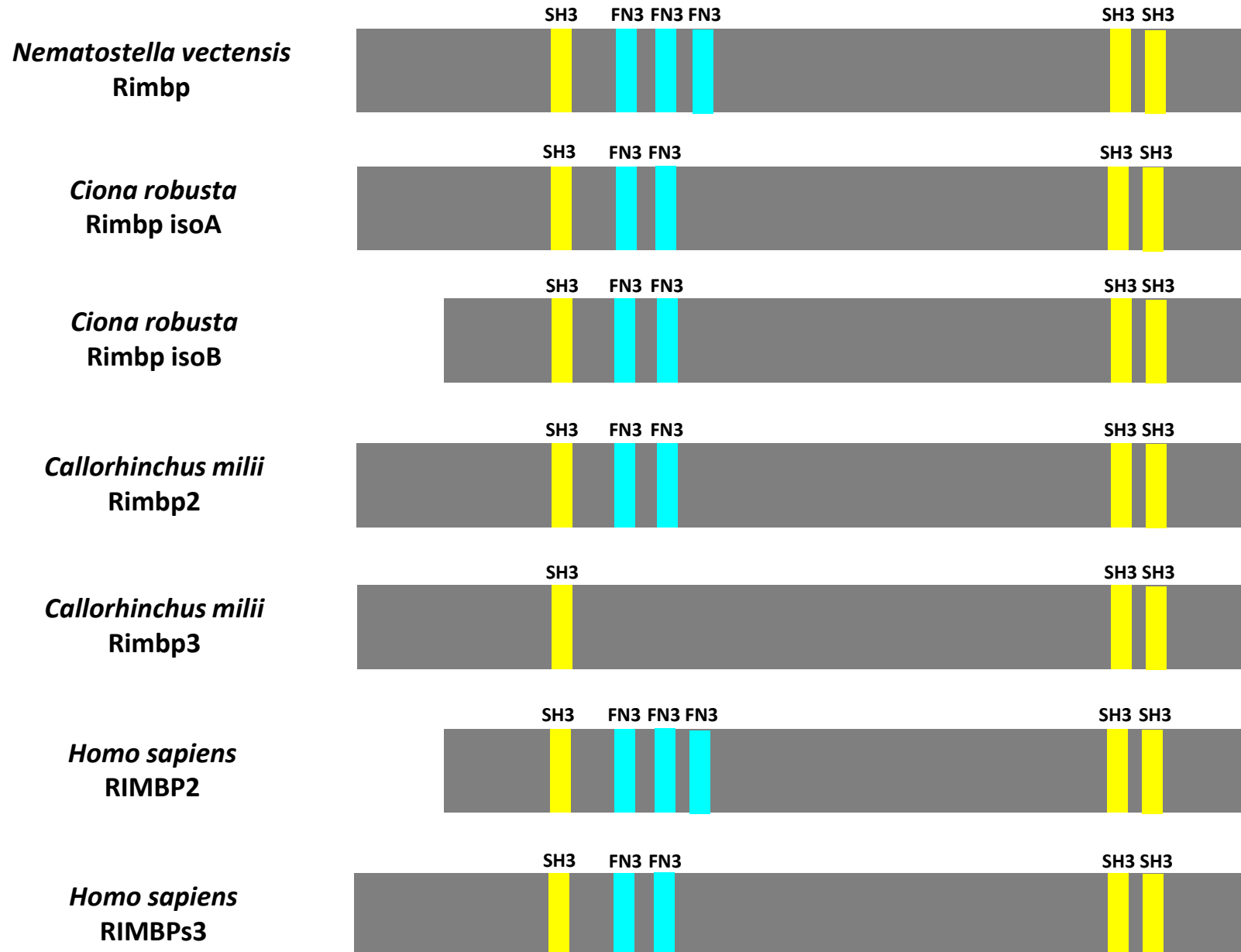

Supplement: Supplementary file 1 [file genes-11-01006-s001.zip › Coppola et al. Figure S1.pdf]

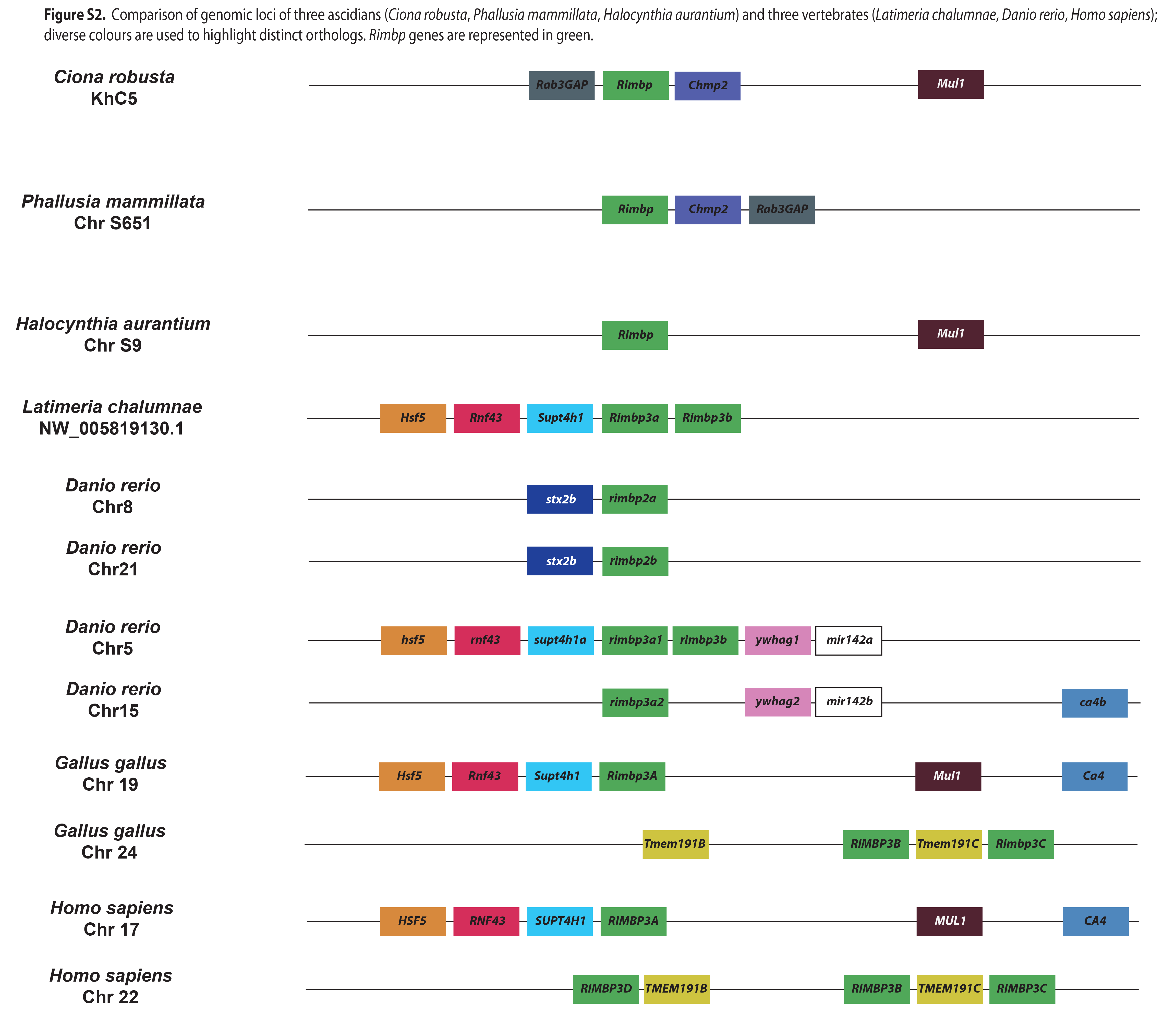

Supplement: Supplementary file 1 [file genes-11-01006-s001.zip › Coppola et al figure S2.tif]
